# Sustainable Dry Machining of Stainless Steel with Microwave-Treated Tungsten Carbide Cutting Tools

**DOI:** 10.3390/mi14061148

**Published:** 2023-05-29

**Authors:** Itemogeng Bernatt Babe, Kapil Gupta, Sujeet Kumar Chaubey

**Affiliations:** Department of Mechanical and Industrial Engineering Technology, University of Johannesburg, Doornfontein Campus, Johannesburg 2028, South Africa; itemogeng9845@gmail.com (I.B.B.); schaubey@uj.ac.za (S.K.C.)

**Keywords:** dry machining, machinability, microwave, stainless-steel, surface roughness, sustainability, tool wear

## Abstract

This paper presents a research investigation conducted on the turning of stainless steel 316 material under a dry environment using microwave-treated cutting tool inserts. Plain tungsten carbide WC tool inserts were exposed to microwave treatment for enhancement of their performance characteristics. It was found that a 20-min microwave treatment resulted in the best tool hardness and metallurgical characteristics. These tool inserts have been used to machine SS 316 material following the Taguchi L_9_ design of experimental techniques. A total of eighteen experiments have been conducted by varying three main machining parameters, i.e., cutting speed, feed rate, and depth of cut, at three levels per parameter. It has been found that tool flank wear increased with all three parameters and surface roughness decreased. At the longest dept of cut, surface roughness increased. An abrasion wear mechanism was found on the tool flank face at a high machining speed and adhesion at low speed. Chips with a helical shape and low serrations have been investigated. Turning SS 316 at optimum machining parameters of 170 m/min cutting speed, 0.2 mm/rev feed rate, and 1 mm depth of cut, as obtained by the multiperformance optimization technique grey relational analysis, resulted in the best values of all machinability indicators: 242.21 µm tool flank wear, 3.81 µm mean roughness depth, and 34,000 mm^3^/min material removal rate, at a single parameter setting. In terms of research achievements, the percentage reduction in surface roughness is approximately 30% and represents an almost ten-fold improvement in the material removal rate. The combination of machining parameters of 70 m/min cutting speed, 0.1 mm/rev feed rate, and 0.5 mm depth of cut is optimum for the lowest value of tool flank wear when considered for single parameter optimization.

## 1. Introduction

In recent years, researchers and engineers have focused on improving the physical and mechanical properties of cutting tool inserts instead of exploring new tool materials. Heat treatment is an important process in altering the material properties by heating and cooling metals under controlled environments [1]. In the metal heat treatment process, temperature control and cooling rate adjustments are used to modify the material’s properties. Tungsten carbide is a widely used cutting tool material in metal cutting due to its suitable mechanical properties, easy availability, and cost-effectiveness [1,2]. 

Nowadays, microwave heat treatment is becoming a popular technology to improve the capabilities of cutting tool inserts by improving mechanical properties such as wear resistance, hardness, and fracture toughness. Microwave heat treatment differs significantly from traditional heat-treatment methods in the sense that the heating is volumetric, selective, and internal [3]. Microwaves whose spectrum lies between the infrared and radio frequency bands are electromagnetic radiations. Electric and magnetic energies are linearly transferred within materials during microwave processing via interfacing interactions between molecules. Microwave technology is widely used for surface modification in terms of coating, glazing, and cladding on various metallic substrate materials for applications such as in communication, aerospace, defense, automotive, and industrial heating [4,5,6].

Stainless steel 316 is one of the most important engineering materials, being exposed to a wide variety of commercial applications. Stainless steel is considered difficult to machine due to its high hardness and low thermal conductivity [7,8]. Before employing the parts and components produced from stainless steel to any application, they undergo extensive machining to obtain a definite shape and size. Conventional machining with plain tungsten carbide tools presents significant challenges, including a high cutting fluid consumption, frequent tool resharpening, extensive wear, and deterioration of work surface quality. Currently, due to strict environmental regulations, minimizing the consumption of cutting fluids is an important aspect. Approximately 15% of the total manufacturing cost occurs from the storage, utilization, and disposal of the cutting fluids. Dry and near-dry machining technologies have been developed and explored for sustainable machining of a wide range of engineering materials. As a sustainable machining technique, dry machining provides alternate solutions by eliminating cutting fluids in the machining process. The effectiveness of dry machining can be improved by utilizing coated, treated, and textured tools in place of plain carbide tools. Previous attempts at the machining of stainless steel and other materials using such techniques are discussed below. 

Jhodkar et al. carried out a detailed investigation on the effectiveness of microwave treatment on tool performance while machining EN8 steel [1,2]. They found a better surface roughness on the machined workpiece and a reduced tool flank and crater wear when machined by treated carbide inserts compared to untreated inserts. The optimization of machining parameters resulted in a 620 rpm cutting speed, 0.1 mm/rev feed, and 0.55 mm depth of cut for the lowest values of tool wear, cutting force, and surface roughness. Both abrasion and adhesion wear mechanisms were observed for microwave-treated tools, but with a lower severity than the untreated tool. Smooth and shiny chips with less serration were found corresponding to microwave tool insert machining. Jadam et al. investigated a substantial decrease in tool wear when using microwave WC-Co tool inserts for the machining of the Inconel 718 alloy [6]. They tested and reported the improved hardness and wear characteristics of the microwave-treated tools. Adhesion and abrasion were found as the dominant wear mechanisms. Mashinini et al. conducted dry machining of SS 316 with textured tools and determined the feed rate as the most influential parameter [9]. They also reported that textured tools are better than plain tools. They successfully optimized the machining parameters and secured the best values of the machinability indicators as R_a_—3.436 μm, MRR—105,187 mm^3^/min average, flank wear—234.63 μm. Another important study reported by Sharma and Gupta is based on the machining of SS304 using coated carbide tools [10]. They successfully obtained a 25% reduction in tool wear and a 15% reduction in average roughness while machining with the use of coated tools than uncoated tools. They recommend the use of multilayer coatings for a longer tool life and better machinability, especially when machining under dry conditions. 

From a detailed review of the important past attempts, it can be summarized that tool inserts with treatment, texture, and coatings can efficiently machine difficult-to-cut materials. Moreover, microwave-treated tools possess the capability to outperform plain tools and enhance machinability to a great extent. Previous attempts on the microwave-treated tool based machining of engineering materials, including stainless steel, are scarce and necessitate future investigations. The present study aims to fill such gaps by investigating the microwave-treated cutting tool based sustainable dry machining of stainless steel 316. It includes a detailed investigation on microwave tool treatment, the effect of cutting parameters on machinability indicators such as flank wear, the surface roughness of the SS 316 workpiece, and the material removal rate, and process optimization. 

## 2. Materials and Methods

The chemical composition of the stainless steel 316 (SS 316) work material is C: 0.08%, Cr: 18%, Ni: 14%, Si: 1%, P: 0.045%, the rest is iron. Its tensile strength and hardness were 480–620 MPa, 215 HV. The SS 316 bar with an initial diameter of 60 mm and a length of 400 mm was used, and the manual lathe Colchester Mascot 1600 UK was used as a machine tool for the turning operation. The microwave-heat-treated tungsten carbide cutting tool inserts equipped with hardness in the range of 2110–2127 HV were used for turning in dry environment. The percentage elemental composition for bare untreated tool insert is: carbide, 7.8%; Co, 9.6%; WC, 82.6% [10].

### 2.1. Microwave Heat Treatment

In microwave heat treatment, the motions of ions and dipoles induces internal heating due to which WC grains are bound by liquified cobalt binders [2]. The heating from the inner core to the surface leads to the development of a skeleton matrix that eventually reduces the voids. Such a structure provides an extra strength to the cutting tool insert by improving hardness, wear characteristics, and fatigue strength. The dielectric properties of the microwave absorption materials used during treatment generate volumetric heating, which results in a temperature rise at a certain penetration depth. 

**Figure 1:**
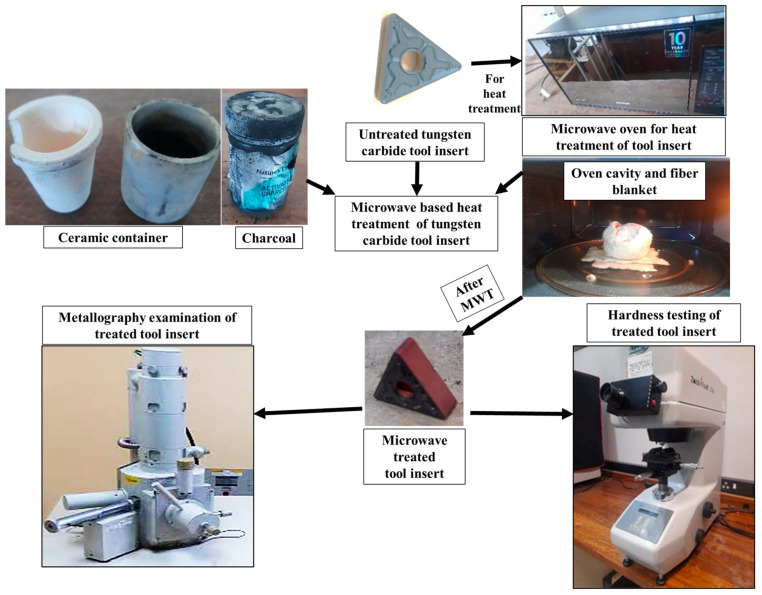
Tasks performed during microwave heat treatment of tungsten carbide tool inserts.

Figure 1 illustrates the instruments used and tasks performed during the microwave heat treatment of tool inserts in the present work. In this study, tungsten carbide (WC) tool inserts were subjected to microwave heat treatment for an effective microwave coupling duration ranging from 10 to 30 min. For this treatment, a microwave oven with 775 Watts of power and a frequency of 2.45 GHz was used. The tool inserts were kept inside a ceramic container possessing activated charcoal for microwave absorption.

The changes in the microstructural and mechanical properties of the microwave-heat-treated tool inserts were evaluated with the help of SEM and Vickers’s hardness test. 

### 2.2. Experimentation and Measurement

**Figure 2:**
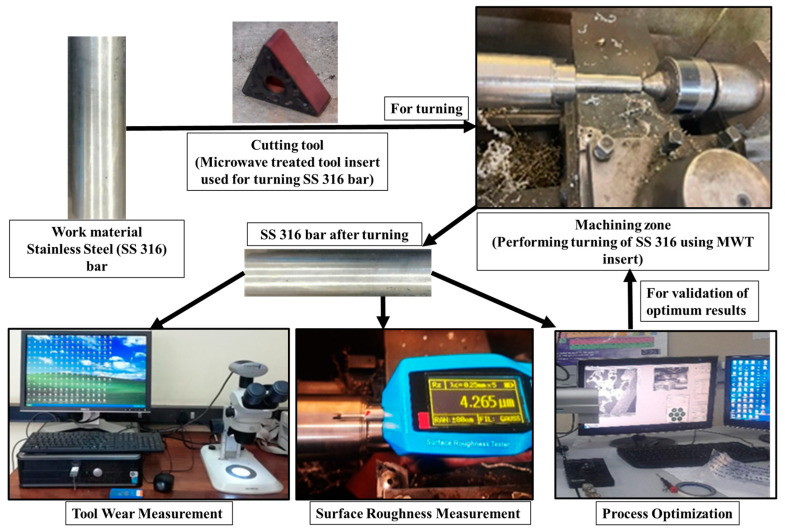
Sequence of tasks performed during machining of SS 316 using MWT tool inserts.

Based upon the selection of three input turning parameters, namely cutting speed ‘*C_S_*’, feed ‘*f*’, and depth of cut ‘*DoC*’ at three levels per parameter, Taguchi’s L_9_ (3^3^) orthogonal array design approach for the design of experiments (DoE) was employed to conduct the experiments. A total of nine turning combinations were designed and experiments were conducted with two replicates for each run. Thus, a total of 18 experimental runs were conducted by performing a turning operation on a SS 316 round bar using a microwave-treated (MWT) WC tool insert. The selection of turning input parameters and their levels, as presented in Table 1, was based on machine constraints, a literature review, and trial experiments. Figure 2 shows the sequences of tasks performed during the machining of SS 316 using the MWT tool insert.

Tool wear, surface roughness, and the material removal rate are the considered machinability indicators used to evaluate the success of microwave-treated-tool based machining of SS 316. The tool wear investigation was conducted on the flank face of the inserts. ISO recommendations were considered, in which the tool life ends at a flank wear value >600 microns. Flank wear and chip morphology were investigated using a Tescan VEGA3 scanning electron microscope (SEM). The mean roughness depth ‘*R_Z_*’ is one of the most important roughness parameters and has been considered as a response/machinability indicator to indicate the quality of the machined surface after turning. The mean roughness depth is superior to the average roughness and maximum roughness in the sense that it gives a real indication of the surface irregularity, since it is an average of the maximum roughness (distance between the highest peak and deepest valley) values of each of the five equal segments of the evaluation length. A TMTecK TMR200 tester was used to measure the *R_Z_* of the turned surfaces after each experimental run.

The volumetric material removal rate ‘*VMRR*’ indicates the removal of materials in mm^3^/min from the workpiece after machining. It is defined as the volume of material removed during the machining process to the total time taken for machining, and it is expressed by the following Equation (1):

(1) $$VMRR=\frac{Weight\;of\;material\;loss\;\left(W_{L}\right)\;during\;machining}{Material\;density\;\left(\rho \right)\;\times \;Total\;machining\;time\;(t)}\left(\frac{{mm}^{3}}{min}\right)$$

where ${W_{L}=W}_{before}-W_{after}$.

Here, *W_before_*_,_ and *W_after_* indicate the weight of the SS 316 round bar before and after turning respectively, *ρ* stands for the density of the SS 316 round bar, and *t* stands for the total machining time (fixed at 330 s) for turning the SS 316 bar for each experimental run.

## 3. Results and Discussion

Table 2 presents the combinations of turning input parameters for the nine experimental runs designed and the corresponding values of the obtained machinability indicators. Two replications were conducted and the average values of the responses are considered for analysis. In this study, the Taguchi L_9_ (3^3^) orthogonal array was employed to identify the influence of the cutting speed ‘*C_S_*’ (70–170 m/min), feed rate ‘*f*’ (0.1–0.2 mm/rev), and depth of cut ‘*DoC*’ (0.5–1.5 mm) on flank wear ‘*F_W_*’ in µm; the mean roughness depth ‘*R_Z_*’ in µm; and the volumetric material removal rate ‘*VMRR*’ in mm^3^/min. The turning experiments on the SS 316 round bar of 60-mm diameter were conducted using microwave-heat-treated tungsten carbide inserts with hardness values in the range of 2110–2128 HV.

### 3.1. Results of Microwave Heat Treatment

**Figure 3:**
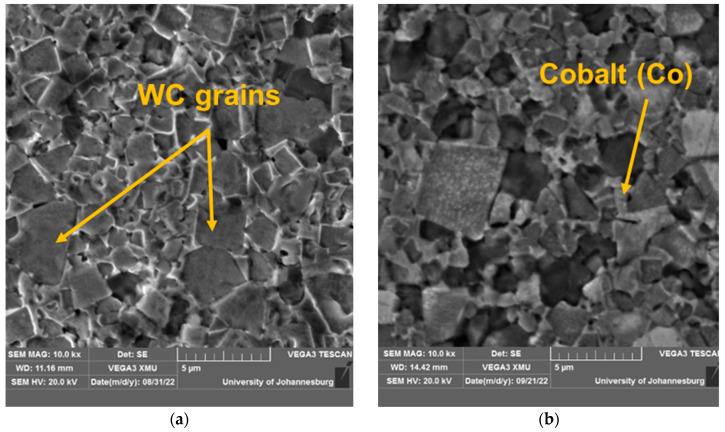

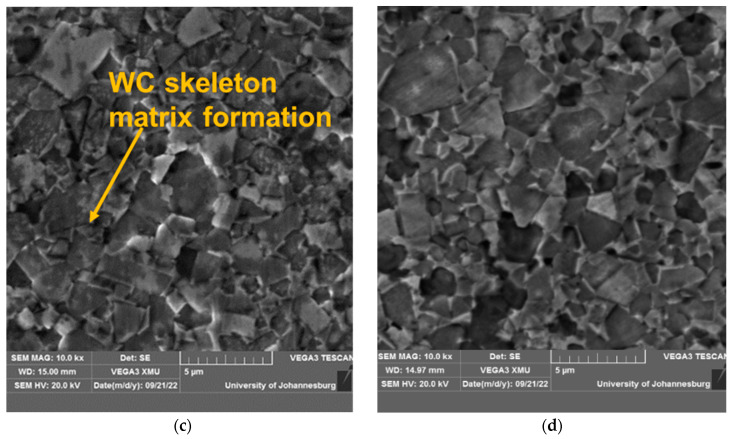
Typical SEM images of cutting tool inserts: (**a**) untreated; (**b**) 10-min treated; (**c**) 20-min treated; and (**d**) 30-min treated.

**Figure 4:**
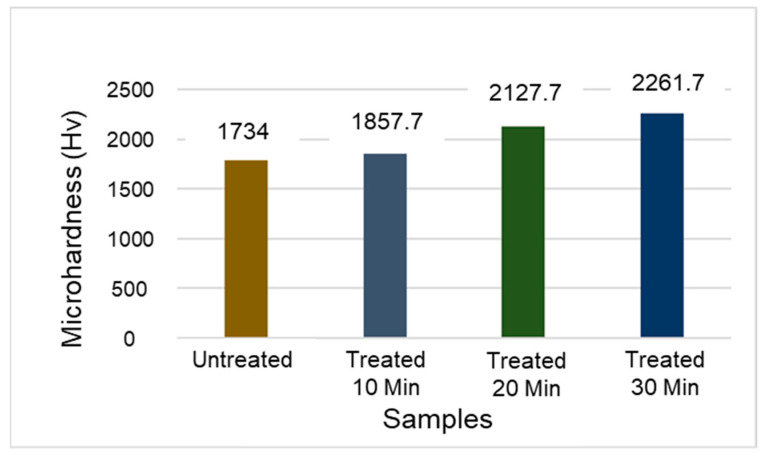
Variation in hardness for different WC tool inserts.

This section presents the results of the microwave heat treatment of the WC tool inserts. Table 3 summarizes the results and Figure 3a–d shows the scanned electron microscopy images of the treated inserts. Figure 3a shows a typical SEM image of an untreated sintered insert, while Figure 3b–d show SEM images of microwave-treated inserts for 10-, 20-, and 30-min time durations, respectively. Figure 4 presents the corresponding values of microhardness for the treated and untreated tool inserts.

The cutting tool insert treated for 20 min was found to be superior from the microhardness value and metallurgical point of view. The SEM image of this tool as shown in Figure 3c shows the formation of a WC skeleton matrix with the binding of WC atoms by liquified cobalt such that a more dense structure is formed. The corresponding microhardness of the tool insert is 2127.7 HV. From the point of view of structure and matrix formation, this 20-min-treated tool has been identified more clearly than the tool inserts treated for 10 (Figure 3b) and 30 (Figure 3d) minutes. All of the tool inserts treated for 20 min have been used for experimentation, and the microhardness value ranges between 2110 and 2128 HV. As shown in Figure 3a, the untreated tool insert is comparatively less dense and with a high porosity.

### 3.2. Effects of Machining Parameters on Machinability Indicators

**Figure 5:**
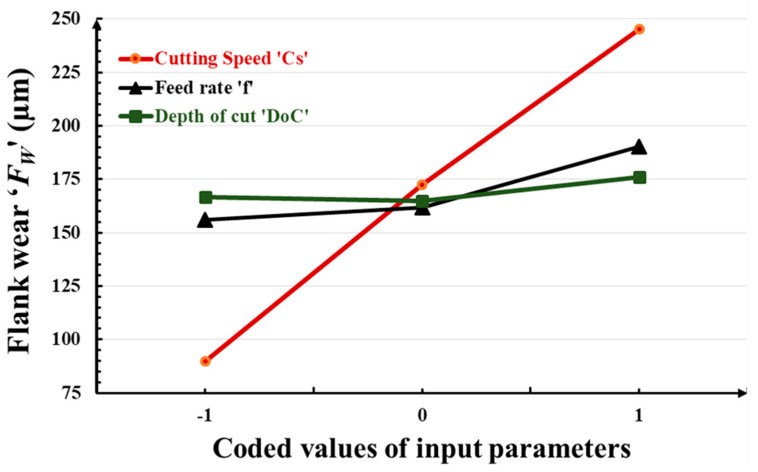
Variation in flank wear ‘*F_W_*’ with input turning parameters.

**Figure 6:**
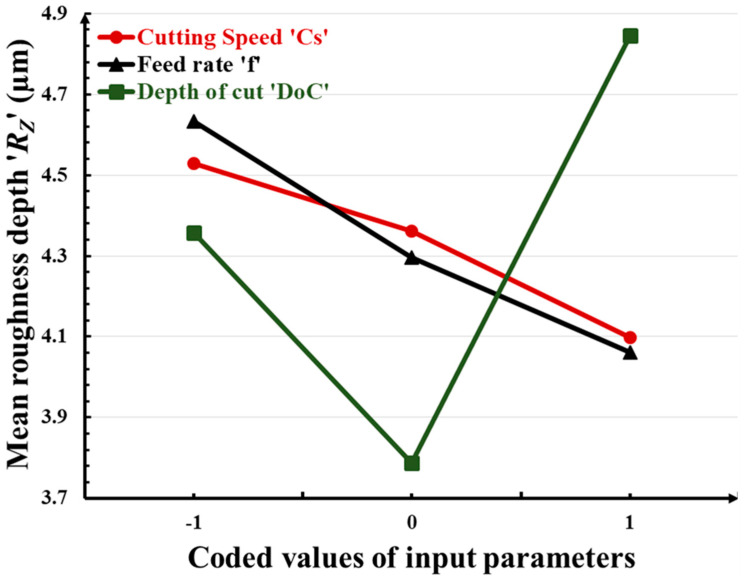
Variation in mean roughness depth ‘*R_Z_*’ with input turning parameters.

**Figure 7:**
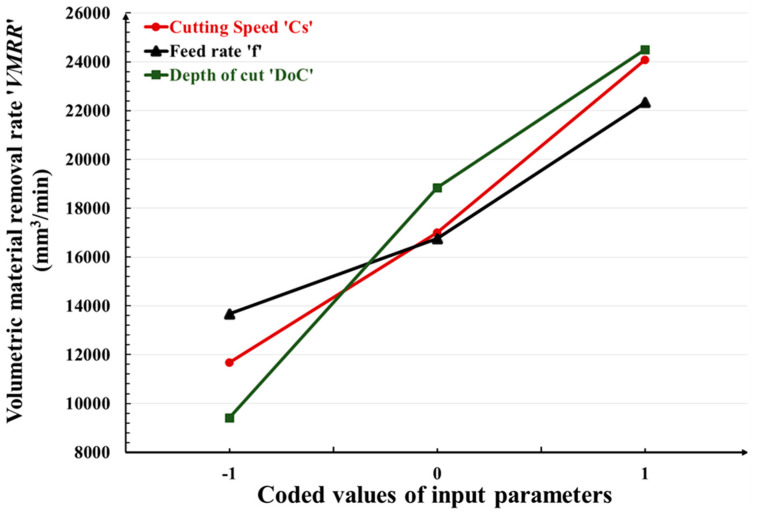
Variation in volumetric material removal rate ‘*VMRR*’with input turning parameters.

In this section, the effects of turning input parameters, namely the cutting speed ‘*C_S_*’, feed rate ‘*f*’, and depth of cut ‘*DoC*’ on flank wear ‘*F_W_*’; the mean roughness depth ‘*R_Z_*’; and the volumetric material removal rate ‘*VMRR*’, are discussed with the help of graphical representation as shown in Figure 5, Figure 6 and Figure 7. In these graphs, the abscissa illustrates the values of the considered machinability indictors/responses, whereas the ordinate indicates the values of the input turning parameters. Increasing the cutting speed, feed rate, and depth of cut leads to an increase in flank wear. The effect of cutting speed is more prominent and a rapid increase in flank wear is observed. The mean roughness depth decreases with high cutting speed and feed rate, whereas it reaches its highest value with the increase of *DoC*. Increasing the cutting speed and feed rate leads to a vanishing of the buildup edges, which consequently ensures smoother surface generation with less rubbing during chip-sliding action. Beyond a *DoC* value of 1.0, the roughness increases due to excessive friction between the tool work interface. It is obvious that high values of all machining parameters cause enhancement in the material removal rate. Higher values of cutting speed increase the rotational speed of the workpiece and higher values of feed increase the movement of the tool insert along the axis of the workpiece, thus increasing the amount of material removal from the workpiece during machining. In contrast, higher values of the depth of cut increase the thickness of the chip and thus remove more materials from the workpiece during machining.

## 4. Optimization

The study of the effects of machining parameters on machinability indicators itself is not enough to measure the success of any machining operation. Optimization of the machining parameters employing a suitable technique is essential to obtain the best machinability indicators [11]. In this work, the grey relational analysis (GRA) statistical technique was used for this purpose [12]. After normalizing the experimental results, the following equations were used in GRA based optimization:

For minimizing the mean roughness depth and flank wear (lower-the-better)

(2) $$Z_{ij}=\frac{\max\left(Y_{i}\right)-Y_{ij}}{\max\left(Y_{i}\right)-\min\left(Y_{i}\right)}$$

For maximizing the material removal rate (higher-the-better)

(3) $$Z_{ij}=\frac{Y_{ij}-\min\left(Y_{i}\right)}{\max\left(Y_{i}\right)-\min\left(Y_{i}\right)}$$

where *max*(*Y_i_*) and *min*(*Y_i_*) are the highest and lowest values of the *i*th response among nine experimental runs as given in Table 2. Further, for the *i*th response of the *j*th experiment, grey coefficient ‘*GC_ij_’* values were obtained using Equation (4).

(4) $${GC}_{ij}=\frac{\Delta_{\min}+\alpha \Delta_{\max}}{\Delta_{ij}+\alpha \Delta_{max}}$$

where ‘α*’* refers to the distinguishing coefficient which has a 0 to 1 range of values. It is usually assigned 0.5 for giving equal weightage to all considered responses. ‘Δ*_ij_’* indicates the inclination of the *i*th response of the *j*th experimental run from its anticipated value. It is equivalent to the absolute variation between the ideal normalized value ‘*Z_ii_’* and the normalized value ‘*Z_ij_’* of the *i*th response, i.e., ${∆}_{ij}=\left|Z_{ii}-Z_{ij}\right|$. In addition, Δ*_min_* and Δ*_max_* are the minimum and maximum values of Δ*_ij_*. The grey relational grade (GRG) of the *j*th experiment ‘*G_j_*’ is computed by considering the average values of the grey coefficient ‘*GC_ij_’* of all of the responses corresponding to the *j*th experimental run in Equation (5).

(5) $$G_{j}=\frac{1}{n}\sum_{i=1}^{n}{GC}_{ij}$$

where ‘*n*’ is the total number of responses.

For GRA-based multiobjective/performance optimization, Table A1 (Appendix A) depicts the values of the grey relational coefficients, as well as the ranks of all experiments undertaken. The best parameter (likely to be the optimum) combination is shown by the highest recorded grey relational grade *G_j_*. For multiperformance GRA-based optimization, the optimum parameter combination is obtained from the third experiment with the following machining parameter combination: cutting speed 170 m/min, feed rate 0.2 mm/rev, and depth of cut 1.0 mm. Table 4 presents the input parameter combination corresponding to the highest grey relational grade and the results of the confirmation experiments. It was found that with the optimum machining settings (i.e., cutting speed 170 m/min, feed 0.2 mm/rev, and depth of cut 1.0 mm), a surface roughness of 3.81 µm and a tool wear of 242.21 µm were obtained. The values of responses obtained after conducting the confirmation experiments are in close agreement with the GRA predictions. The multiperformance optimization, certainly, did not provide the lowest value of tool wear. As presented in Table 2, the lowest tool wear can be obtained at a 70 m/min cutting speed, 0.1 mm/rev feed rate, and 0.5 mm depth of cut. However, an approximately 30% reduction in mean roughness depth was achieved when comparing the results of multiperformance optimization and the maximum value of mean roughness depth (5.38 µm) corresponding to Experiment 1 in Table 2. Similarly, an almost ten-fold improvement in the material removal rate was achieved if we compare the value obtained during multiperformance optimization and its lowest value (3500 mm^3^/min) corresponding to Experiment 1 in Table 2.

## 5. Study of Tool Wear and Chip Morphology

**Figure 8:**
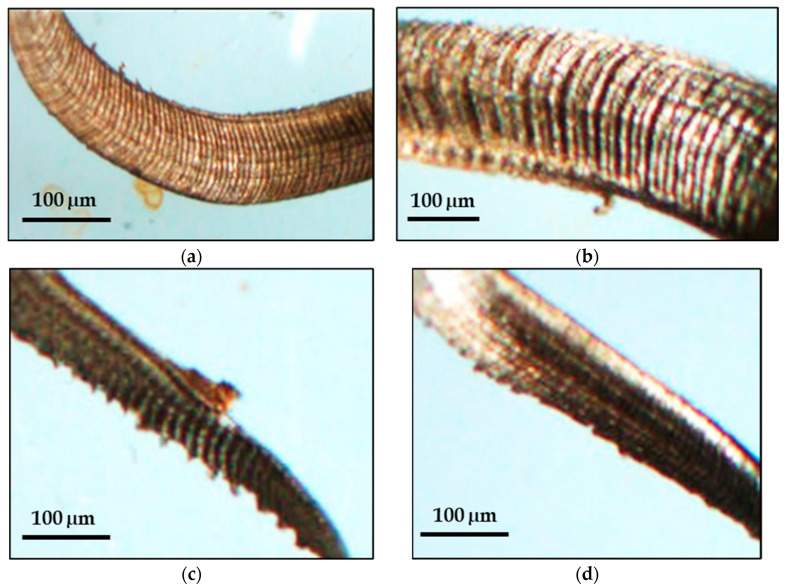
Chip morphology at (**a**) *C_S_*: 70 m/min; *f*: 0.1 mm/rev; *DoC*: 0.5 mm; (**b**) *C_S_*: 120 m/min; *f*: 0.1 mm/rev; *DoC*: 1 mm, (**c**) *C_S_*: 170 m/min; *f*: 0.2 mm/rev; *DoC*: 1 mm, (**d**) confirmation experiment.

**Figure 9:**
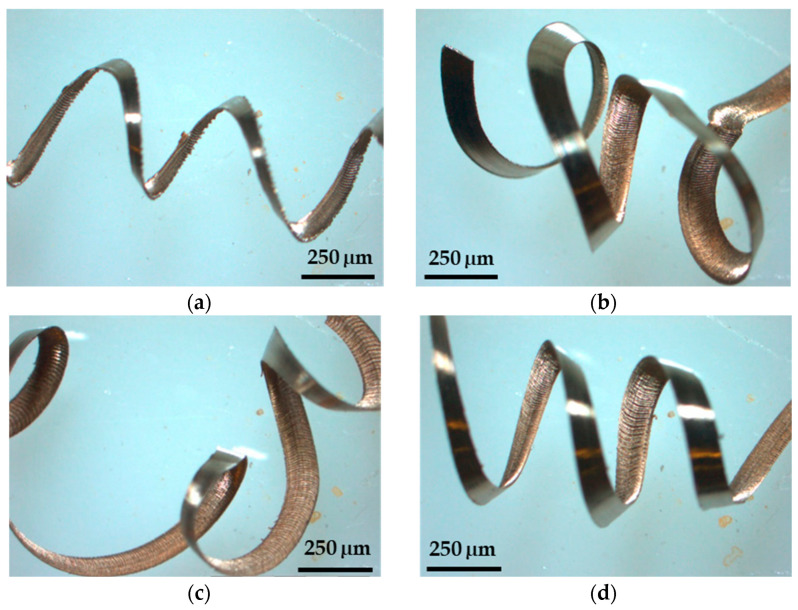
Chip morphology at (**a**) *C_S_*: 70 m/min; *f:* 0.1 mm/rev; *DoC*: 0.5 mm, (**b**) *C_S_*: 120 m/min; *f:* 0.1 mm/rev; *DoC*: 1 mm, (**c**) *C_S_*: 170 m/min; *f*: 0.2 mm/rev; *DoC*: 1 mm, (**d**) confirmation experiment.

Machining chips are divided into three types based on their morphology: aperiodic saw-tooth chips, continuous chips, and periodic saw-tooth chips [13,14]. The primary turning factors, such as cutting speed, feed rate, depth of cut, the shape of tool inserts, and insert material, all have a substantial impact on chip formation. In the current investigation, SS 316 was machined under dry conditions. Considering the optical micrographs (Figure 8a–d) and actual pictures (Figure 9a–d) of the chips obtained with different combinations of machining parameters, it was observed that the thicker chips were found in the event of 120 m/min and 170 m/min using a 20-min microwave-treated carbide insert due to the high coefficient of friction between the tool–chip edge. The outside circle had an irregular shape and ductile chip tearing as the cutting speed increased. The chip segmentation became more visible with the increase in cutting speed. More chips were burnt due to the high cutting temperature, resulting in shorter chips and even fragmentation when cut at a speed greater than 70 m/min. The friction between the tool rake face and the chip, as well as the degree of plastic deformation and heat formation, are all affected by the cutting speed. The back surface of chips is generally in the secondary deformation zone during machining, and the cutting speed has a considerable influence on friction. Figure 9 depicts ribbon and helical tubular type chip formation at almost all levels of cutting speed during treated-tool-based machining of SS 316.

**Figure 10:**
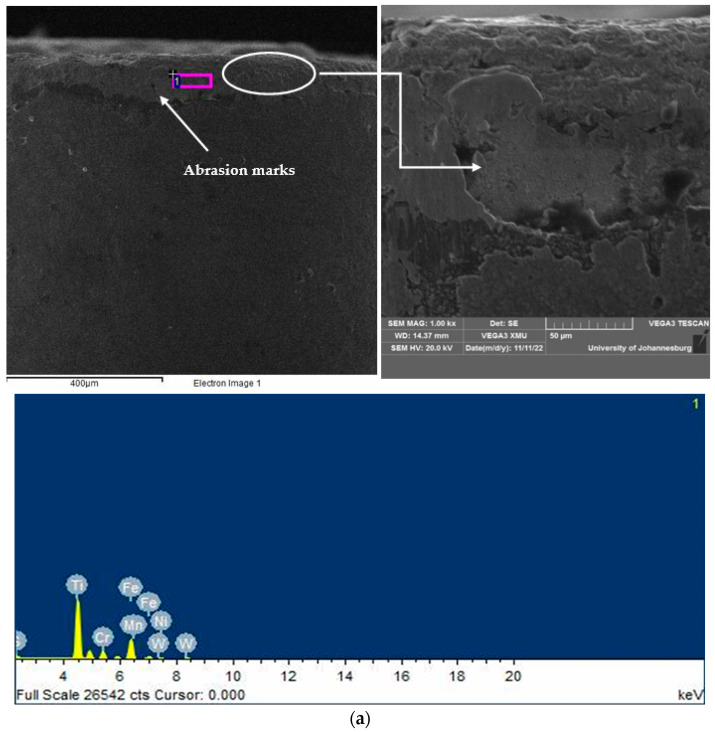

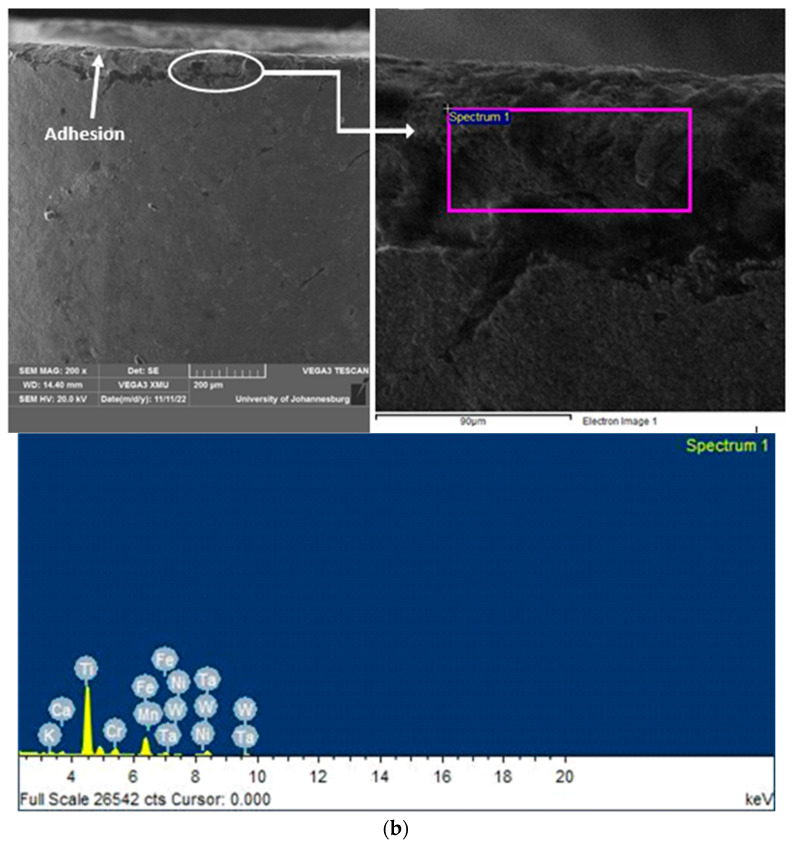
Scanning electron micrographs and energy dispersive X-ray analysis reports of microwave-heat-treated (20 min) tool insert: (**a**) abrasion wear occurs at a high cutting speed, i.e., *C_S_*—170 m/min, *f*—0.2 mm/rev, *DoC*—1 mm; (**b**) adhesion wear occurs at a low cutting speed, i.e., *C_S_*—70 m/min, *f*—0.1 mm/rev, *DoC*—0.5 mm.

Figure 10a illustrates the micrographs obtained from a scanning electron microscope (SEM) and energy dispersive X-ray report to study the wear mechanism of the microwave-heat-treated tool insert used at 170 m/min, 0.2 mm/rev, and 1 mm. Although the predominant mechanism is abrasion, the amount of tool wear is high, i.e., it is at a longer depth on the flank face. In contrast, as evident in Figure 10b, at a low speed (70 m/min), the wear mechanism is adhesion but does not extend up to a long depth on the flank face of the cutting tool insert. 

## 6. Conclusions

In this research, plain tungsten carbide cutting tool inserts have been successfully treated by microwave to improve their cutting performance and utilized to turn the SS 316 material under a dry environment. A detailed investigation on turning SS 316 with microwave-treated tool inserts under a dry environment was performed, and machining parameter optimization was conducted to secure the best values of the considered machinability indicators of cutting speed, feed rate, and depth of cut. The following conclusions can be drawn from this investigation:

- The microwave treatment caused the formation of a WC skeleton matrix that provided a favorable microstructure and extra hardness to the cutting tool inserts.
- The tool inserts which underwent microwave treatment for 20 min resulted in the best metallurgical characteristics, with an improved hardness ranging between 2110 and 2128 HV, suitable for turning SS 316.
- High flank wear has been observed with increasing cutting speed, feed rate, and depth of cut.
- The mean roughness depth decreased with increases in speed and feed rate, but increased at a high depth of cut.
- A GRA-based multiperformance optimization results in values of 242.21 µm for tool flank wear, 3.81 µm for the mean roughness depth, and 34,000 mm^3^/min for the material removal rate, while machining SS 316 at the optimum machining parameters of 170 m/min cutting speed, 0.2 mm/rev feed rate, and 1 mm depth of cut.
- Helical tubular type thick chip formation with fewer serrations takes place when machining at cutting speeds higher than 70 m/min. Flank wear occurred at higher speeds due to abrasion, whereas adhesion is observed for lower speed machining.

Consequently, it is recommended that machining stainless steel (SS 316) material in a dry environment can result in better machinability if machined at a higher speed and feed rate and with a moderate value of depth-of-cut, using microwave-treated tools.

## Figures and Tables

**Table 1:** Details of machining parameters for microwave turning of SS 316.

Input Turning Parameters		Levels			Responses
Coded Name	Actual Name with Their ‘*Abbreviation*’	L_1_	L_2_	L_3_	
A	Cutting Speed ‘*CS*’ (m/min)	70	120	170	Tool wear: Flank wear (µm) Surface roughness: Mean roughness depth ‘*R_Z_*’ (µm) Material removal rate: Volumetric material removal rate ‘MRR’ (mm^3^/min)
B	Feed Rate ‘*f*’ (mm/rev)	0.1	0.15	0.2	
C	Depth of Cut ‘*DoC*’ (mm)	0.5	1	1.5	

**Table 2:** Experimental combinations and corresponding values of the machinability indicators for MWT tools-based turning of SS 316.

Expt. No.	Input Turning Parameters			Machinability Indicators								
				Flank Wear ‘*F_W_*’ (µm)			Mean Roughness Depth ‘*R_Z_*’ (µm)			Volumetric Material Removal Rate ‘*MRR*’ (mm^3^/min)		
	Cutting Speed ‘C_S_’ (m/min)	Feed ‘*f*’ (mm/rev)	Depth of Cut ‘*DoC*’ (mm)	*R*_1_	*R*_2_	Mean	*R*_1_	*R*_2_	Mean	*R*_1_	*R*_2_	Mean
**1**	**70**	**0.1**	**0.5**	72.56	69.71	**71.13**	5.46	5.31	**5.38**	3500	3500	**3500**
2	120	0.15	1.5	157.63	179.93	168.78	6.52	4.21	5.37	27,000	27,000	27,000
**3**	**170**	**0.2**	**1**	269.90	247.67	**258.78**	3.00	4.61	**3.81**	34,000	34,000	**34,000**
4	70	0.15	1	83.04	71.45	77.24	4.26	3.78	4.02	10,500	10,500	10,500
5	120	0.2	0.5	193.77	186.85	190.31	4.95	3.42	4.19	12,000	12,000	12,000
6	170	0.1	1.5	224.91	251.78	238.34	5.94	4.02	4.98	25,500	25,500	25,500
7	70	0.2	1.5	131.49	110.73	121.11	3.58	4.79	4.19	21,000	21,000	21,000
8	120	0.1	1	173.01	143.33	158.17	3.50	3.57	3.53	12,000	12,000	12,000
9	170	0.15	0.5	242.21	235.29	238.75	3.95	3.05	3.50	12,750	12,750	12,750

**Table 3:** Microhardness results for microwave-heat-treated cutting tool inserts.

Measurement	Plain WC Tool Insert	Microwave-Treated WC Tool Insert		
		Treatment Time (min)		
		10	20	30
Microhardness (HV)	1734	1857.7	2127.7	2261.7

**Table 4:** Results of GRA-based optimization and confirmation experiments.

Machining Details		Optimized Value by GRA	Confirmation Results
			Average of R_1_ and R_2_
Turning process parameters	Cutting speed ‘*C_S_’* (mm/min)	170	170
	Feed rate ‘*f*’ (mm)	0.2	0.2
	Depth-of-cut ‘*DoC*’ (mm)	1.0	1.0
Responses	Flank wear ‘*F_W_*’ (µm)	258.78	242.21
	Mean roughness depth ‘*R_Z_*’ (µm)	3.81	3.81
	Vol. material removal rate ‘*VMRR*’ (mm^3^/min)	34,000	34,000

## Data Availability

The research data will be made available upon request.

## Appendix A

**Table A1:** Results of grey relational optimization.

Expt. No.	Responses			Normalized Values			Deviation Sequences			Grey Relational Coefficient			GRG	Rank
	*F_W_*	*R_Z_*	MRR	*F_W_*	*R_Z_*	MRR	*F_W_*	*R_Z_*	MRR	*F_W_*	*R_Z_*	MRR		
1	71.13	5.38	3500	1.00	0.000	0.00	0.00	1.00	1.00	1.00	0.33	0.33	0.56	6
2	168.78	5.37	27,000	0.48	0.008	0.77	0.52	0.99	0.23	0.49	0.33	0.68	0.50	7
**3**	**258.79**	**3.81**	**34,000**	**0.00**	**0.84**	**1.00**	**1.00**	**0.16**	**0.00**	**0.33**	**0.75**	**1.00**	**0.70**	**1**
4	77.24	4.02	10,500	0.97	0.72	0.23	0.03	0.27	0.77	0.94	0.64	0.39	0.66	2
5	190.31	4.19	12,000	0.37	0.64	0.28	0.64	0.36	0.72	0.44	0.58	0.41	0.48	8
6	238.35	4.98	25,500	0.11	0.21	0.72	0.89	0.79	0.28	0.36	0.39	0.64	0.46	9
7	121.11	4.19	21,000	0.73	0.63	0.57	0.27	0.37	0.43	0.65	0.58	0.54	0.59	5
8	158.17	3.53	12,000	0.53	0.98	0.28	0.46	0.02	0.72	0.52	0.97	0.41	0.63	3
9	238.75	3.50	12,750	0.11	1.00	0.30	0.89	0.00	0.70	0.36	1.00	0.42	0.59	4
